# Hyperbaric oxygen treatment for late radiation-induced tissue toxicity in treated gynaecological cancer patients: a systematic review

**DOI:** 10.1186/s13014-022-02067-6

**Published:** 2022-10-06

**Authors:** Nadine I. Geldof, Rob A. van Hulst, Milan L. Ridderikhof, David N. Teguh

**Affiliations:** Department of Surgery, Hyperbaric Medicine, Amsterdam UMC, location AMC, Meibergdreef 9, 1105 AZ Amsterdam, The Netherlands

**Keywords:** Gynaecological cancers, Hyperbaric oxygen therapy, Tissue toxicity, Systematic review, Radiation oncology

## Abstract

**Purpose:**

The aim of this study was to investigate the result of hyperbaric oxygen therapy (HBOT) in women with treated gynaecological malignancies who suffer from late radiation-induced tissue toxicity (LRITT). Moreover, which symptoms of LRITT benefit most from HBOT was evaluated as well.

**Material and Methods:**

An online literature search was conducted using PubMed; Embase and the Cochrane Library. Studies were included if the study examined gynaecological cancer patients who had been treated with radiotherapy, who suffered from LRITT and who subsequently received HBOT. In addition, the outcome measures were based on examining the effects of HBOT.

**Results:**

Twenty-one articles were included. The study investigating proctitis reported an improvement and three out of four studies investigating cystitis reported decreased complaints in women treated for gynaecological malignancies. In addition, all studies reported improvement in patients with wound complications and fifty percent of the studies reported better Patient Reported Outcome Measurements (PROMS) in women with gynaecological malignancies. Finally, all studies, except one related to pelvic malignancies reported reduced prevalence of symptoms for cystitis and proctitis and all studies reported better PROMS. However, only eleven studies reported p-values, nine of which were significant.

**Conclusion:**

This study demonstrated that HBOT has a positive effect in women with gynaecological LRITT. Within the included patient group, gynaecological cancer patients with wound complications seem to benefit most from this treatment compared to other late side effects of LRITT.

**Supplementary Information:**

The online version contains supplementary material available at 10.1186/s13014-022-02067-6.

## Introduction

Worldwide, nearly 1.4 million women were diagnosed with gynaecological cancers in 2020 [1]. Due to aging populations and population growth in developed countries, this number will increase every year [2].

The gynaecological cancers included in this review are ovarian-, cervical-, vaginal-, uterine- and vulvar cancers. Gynaecological cancer patients are often treated using multimodality therapies, including surgery and radiotherapy or chemotherapy. Only in early stage gynaecological cancer patients, surgical treatment without adjuvant therapy is often sufficient [3–6]. In addition, radiotherapy is the standard treatment of vaginal cancer, as the cancer’s proximity to normal tissues limits its surgical options as a treatment for this type of cancer [7]. Radiotherapy as a component for the treatment of gynaecological malignancies has increased the local control of cervical-, uterine-, vulvar- and vaginal malignancies [8]. Although radiotherapy provides an increased chance of local control, it can also cause damage to surrounding organs and tissues [8]. This is referred to as late radiation-induced tissue toxicity (LRITT). LRITT can be described as damage to organs and tissues that occurs at least three months after radiotherapy has ended [9].

LRITT can arise through three underlying mechanisms whereby radiotherapy can damage both parenchymal and vascular cells. The first mechanism of oxidative damage to cells is caused by a local increase of reactive oxygen species (ROS) at the site of the tumor. As a result, these ROS also produce pro-inflammatory cytokines and chemokines that cause inflammation and ultimately tissue damage and cell death. Finally, radiotherapy can cause tissue damage and cell death via the innate immune response. This mechanism involves both bone marrow-derived cells and macrophages [10].

The average annual incidence of LRITT is 13.8% [11]. These late side effects of radiotherapy can be very diverse and present clinically as proctitis, cystitis or as necrosis and poor healing of wounds in the vaginal, vulvar and rectovaginal area.

One of the treatment modalities that could reduce or even cure complaints of LRITT is hyperbaric oxygen therapy (HBOT). LRITT is an internationally accepted indication for HBOT. This treatment implies creating a hyperbaric environment in which patients are being administered high oxygen concentrations. It uses a chamber in which the patient is administered 100% oxygen at a pressure ranging from 2.0 to 3.0 atmospheric pressure (ATA) for a duration of 60 to 120 min. Patients could be treated in monoplace chambers, in which one patient can be treated or multiplace chambers, in which multiple patients can be treated simultaneously [12].

The treatment effects of HBOT in LRITT are based on an increased systemic concentration of reactive oxygen species (ROS) and reactive nitrogen species (RNS), resulting in increased wound growth factors and a mobilization of stromal progenitor cells (SPc) from the bone marrow. As a result of these two processes, increased neovascularization will occur. In addition, the increased systemic concentrations of ROS and RNS cause neutrophil $\upbeta$-actin nitrosylation, reduced monocyte chemokine synthesis and changes in ischemic preconditioning. Subsequently, these processes result in a reduced inflammatory response and an improved post-ischemic tissue survival. All these processes result in a better neovascularization and wound healing. These pathways make HBOT clinically relevant for the treatment of LRITT [13].

The aim of this literature review was to investigate the result of HBOT in women with treated gynaecological malignancies who suffer from LRITT. This systematic review specifically investigated the effect of HBOT on various symptoms, which has not been done in previous studies.

## Material and methods

### Literature search

An online literature search was conducted in PubMed, Embase and the Cochrane Library on August 18, 2021. The included studies were published between 1992 and 2020. The main terms, along with many synonyms, used in the literature search in PubMed were: ‘hyperbaric oxygen therapy’; ‘gynecological’; and various types of gynaecological cancers. The terms used in the literature search in Embase were: ‘gynecological cancer’; ‘female genital tract cancer’; and ‘hyperbaric oxygen therapy’. Finally, the terms ‘hyperbaric oxygenation’; ‘hyperbaric oxygen therapy’; ‘gynecological cancer’ and ‘genital diseases’ were used in the literature search in the Cochrane Library. A detailed overview of the literature searches is described in Additional files 1, 2, 3.

### Outcome measures

For this review, the Late Effects Normal Tissue Task Force–Subjective, Objective, Management, Analytic (LENT—SOMA) score and clinical outcome score were used as primary outcome measures as these were the most commonly used outcome measures in the included studies. The Expanded Prostate Index Composite (EPIC) score, Inflammatory Bowel Disease Questionnaire (IBDQ) rectal bleeding score and many other outcome measures were used as secondary outcome measures. The EPIC score is often used to determine symptoms after radiotherapy in prostate cancer patients but can also be used for other types of pelvic cancers, such as vulvar malignancies. The urology and bowel sections of the EPIC score are not specifically based on symptoms of prostate cancer but answer questions about side effects after radiotherapy in the pelvic region [14].

### Study selection

All articles of the literature search have been screened for title and abstract by the first author. Potentially eligible articles that could be included based on their title and abstract, were screened in full text for further assessment. The heading ‘similar articles’ was searched in PubMed in order to find additional articles.

#### Inclusion criteria

Articles were selected based on multiple inclusion criteria: (1) the patients must have had gynaecological malignancies in the past and had been treated with radiotherapy, (2) the patients must be affected by late radiation-induced tissue toxicity (LRITT) and (3) the patients have been treated with hyperbaric oxygen therapy (HBOT).

#### Exclusion criteria

The exclusion criteria were: (1) the study was not published in English, (2) the study was not performed in humans, (3) articles that were not available, including abstract, were excluded and (4) case reports and case series were excluded.

### Data extraction

Data such as study characteristics, patient characteristics, characteristics of HBOT and outcome measures were extracted. In addition, the time from radiation to injury, the time from injury to treatment and the time to follow-up have been reported. The comparison of the symptoms and the side effects of HBOT have also been extracted. Finally, the results and significance levels, if available, were reported. This also included Patient Reported Outcome Measures (PROMS), in which the quality of life, pain scores and depression symptoms were evaluated.

### Statistical description

Data were presented on a descriptive manner. The outcomes were presented with the p-values, 95% confidence interval (95% CI) or standard deviation (SD). Some studies have used the odds ratio (OR) to present the effect of HBOT. Finally, some studies only reported percentages or the improvement of the symptoms.

## Results

### Study selection

**Fig. 1:**
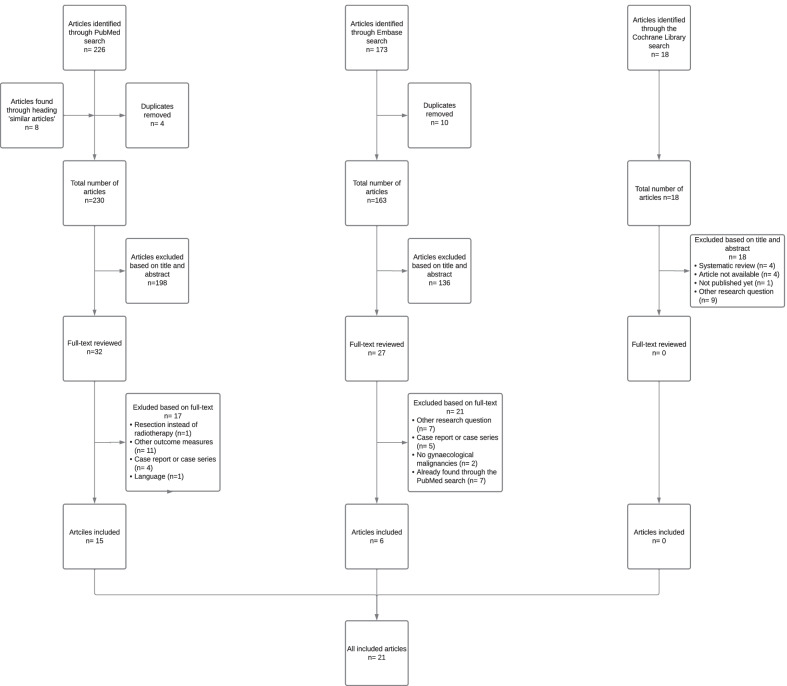
Flow chart of the literature search

We have found 226 articles through the PubMed search. 173 articles were found through the search in Embase and 18 articles were found through the search in the Cochrane Library. Subsequently, 198 articles were excluded from the PubMed search, 136 articles were excluded from the Embase search and 18 articles were excluded from the Cochrane Library search based on title and abstract. For detailed exclusion criteria, see Additional files 4, 5, 6. After this exclusion, 32 articles from the PubMed search were screened for full-text and 27 articles from the Embase search were screened for full-text. After we excluded 17 articles from the PubMed search based on full-text, 15 articles were ultimately included in this review. In the Embase search, 21 articles were excluded based on full-text after which 6 articles were ultimately included in this review. Finally, the total number of included studies was 21 [14–34]. For the detailed flow chart, see Fig. 1.

### Included studies

### Study designs

Of the included 21 studies, different types of research designs were used by the researchers. Four studies were randomized controlled trials, one was a cohort study, twelve studies used a retrospective analysis and two studies used prospective analysis. In addition, one study was a combined retrospective and prospective observational study. One study did not report a study design or the study design could not be inferred. For a detailed overview, see Additional file 7.

### Patient characteristics

A total of 1026 patients were included in these twenty-one studies, of which 531 patients had gynaecological cancers. The age of the patients varied widely with a range of seven to ninety-one years. For a detailed overview, see Table 1.

**Table 1:** Study and patient characteristics

Study, year	Gynaecological patients (total number of pelvic patients)	Mean age (range)	Primary cancer diagnosis	Clinical symptoms	Outcome measures	Chamber	Pressure (ATA)	Sessions (n)	Time (min)
Oscarsson et al., 2013 [14]	2 (39)	71 (35–84)	Prostate (*n* = 34) Rectal (*n* = 3) Cervical (*n* = 2)	Cystitis and proctitis (bleeding from mucosa, pain in the pelvis region, incontinence, frequent and/or imperative urge for defecation and/or urination)	EPIC (urinary and bowel domain)	Multiplace (*n* = 35) Monoplace (*n* = 4)	${2.4}^{\mathrm{a}}$	36 (mean)	90
Glover et al., 2016 [15]	38 (84)	Treatment group 62.3 Control group 62.0	Prostate (*n* = 33) Anus (*n* = 8) Vagina (*n* = 4) Cervix (*n* = 22) Uterus (*n* = 11) Anal canal (n = 1) Vulvar (*n* = 1) Retroperitoneum (n = 1) Pelvis (*n* = 1) Rectum (*n* = 1) Bladder (*n* = 1)	Chronic bowel dysfunction (grade 1 or 2 gastrointestinal symptoms)	Bowel component of IBDQ score, IBDQ rectal bleeding score, LENT-SOMA score, CTCAE scale, EORTC QLQ-C30 questionnaire and QLQ-CR38 module	Monoplace and multiplace	2.4 (treatment group) 1.3 (control group)	${40}^{\mathrm{b}}$	90
Oscarsson et al., 2019 [16]	20 (79)	Treatment group 64.0 (13.6 SD) Control group 64.8 (10.7 SD)	Cervical (*n* = 18) Prostate (*n* = 54) Rectum (*n* = 3) Uterus (*n* = 2) Other (*n* = 2)	Cystitis	EPIC (urinary and bowel), SF-36 score, histological changes in the urinary bladder biopsies and LRMGS grades	Multiplace and monoplace	2.4–2.5	30–40	80—90
Oliai et al., 2012 [17]	2 (15)	69.5 (55–84) *	Prostate (*n* = 12) Cervical (*n* = 1) Vulvar (*n* = 1) Rectal (*n* = 1)	Cystitis	LENT-SOMA score, clinical recurrence and severity of hematuria	Monoplace	2.0	24 (mean) *	90–120
Sidik et al., 2007 [18]	65	Treatment group 47 (± 5.5 SD) Control group 44.7 (± 6.2 SD)	Cervical (*n* = 65)	Overall side effects	LENT-SOMA score and Karnofsky score	NR	2.0–3.0	${>18}^{\mathrm{c}}$	NR
Clarke et al., 2008 [19]	104 (120)	NR	Cervical (*n* = 93) Uterus (*n* = 10) Endometrium (*n* = 1) Prostate (n = 13) Colon (*n* = 1) Rectal (*n* = 2)	Proctitis	LENT-SOMA bowel function, SF-12 general health function survey, clinical evaluation and patients’ beliefs	NR	2.0	${30}^{\mathrm{d}}$	90
Parra et al., 2011 [20]	4 (25)	66.7 (42–80)	Prostate (*n* = 20) Bladder (*n* = 1) Cervical (*n* = 3) Endometrium (*n* = 1)	Cystitis	Clinical response of macroscopic bleeding	Multiplace	2.2	40	90
Rud et al., 2009 [21]	16	NR	Cervix (*n* = 14) Endometrium (*n* = 1) Ovarian (*n* = 1)	Overall tissue injuries (unacceptable pain in pelvic region)	BPI score, MADRS score, MRI, use of pain descriptors or analgesics and clinical changes	NR	2.4	21	90
Safra et al., 2008 [22]	11 (13)	55.2 (32–82) *	Cervix (*n* = 8) Endometrium (*n* = 2) Vagina (*n* = 1) Rectal (*n* = 1) Bladder (*n* = 1)	Cystitis, proctitis, rectovaginal fistulas, vesicovaginal fistulas, vaginal ulcers and wound healing complications	NCI Common Toxicity Criteria and clinical changes of proctitis, cystitis and wound complications	Multiplace	2.0	27 (mean)	90
Jones et al., 2006 [23]	6 (10)	65 (39–79)	Prostate (*n* = 3) Cervix (*n* = 4) Uterus (*n* = 1) Rectum (*n* = 1) Vagina (*n* = 1)	Proctitis	LENT-SOMA score and clinical changes	NR	2.0–2.5	36–41	90
Williams et al., 1992 [24]	13 (14)	53 (35–78)	Cervix (*n* = 9) Endometrium (*n* = 1) Vagina (*n* = 3) Colon (*n* = 1)	Necrotic wounds	Clinical changes of vaginal necrosis and fistula	NR	2.0	44 (mean)	90–120
Feldmeier et al., 1996 [25]	30 (44)	60.9 (33–80) *	Cervix (*n* = 19) Endometrium (*n* = 3) Vulva (*n* = 5) Ovarian (*n* = 2) Uterus (*n* = 1) Prostate (*n* = 1) Testicular (*n* = 2) Rectum/anus (*n* = 4) Bladder (*n* = 2) Ewings sarcoma (*n* = 1) Mycosis fungoides (*n* = 1) Unknown (*n* = 1) Skin (*n* = 1) Urethra (*n* = 1)	Overall injuries (necrotic wounds, fistulas, cystitis, enteritis colitis, caecal perforation, soft tissue and ulcers)	Clinical changes of healing injuries, closure of fistulas and necrotic wounds	Multiplace	2.4	27.2 (mean) *	90
Al-Ali et al., 2010 [26]	3 (14)	Treatment group 73.5 (59–88) * Control group 51*	Prostate (*n* = 10) Colon (*n* = 1) Cervix (*n* = 1) Vulvar (*n* = 1) Uterus (*n* = 1)	Cystitis (macroscopic hematuria)	Clinical changes in hemorrhagic cystitis and bleeding	NR	2.5	30 *	60
Bui et al., 2004 [27]	7 (45)	64 (7–88)	Head and neck (*n* = 31) Pelvic (*n* = 7) Other (*n* = 7)	Overall side effects (osteoradionecrosis, soft tissue necrosis, proctitis and cystitis)	RTOG criteria	NR	2.4	40 (median) *	100
Andren et al., 2020 [28]	7 (52)	67.9 (SD 10.1)	Prostate (*n* = 41) Cervix (*n* = 4) Rectum (*n* = 3) Endometrium (*n* = 2) Bladder (*n* = 1) Vulvar (*n* = 1)	Proctitis and cystitis	LENT-SOMA score (bladder and bowel domain)	Multiplace and monoplace	2.4 (multiplace) 2.0 (monoplace)	31 (mean)	${90}^{\mathrm{ e}}$
Ngoo et al., 2018 [29]	(18)	$${\begin{array}{c}59.5 \\ (42-82)\end{array}}^{\mathrm{f}}$$	NR	Cystitis	Clinical	NR	2.4	27 (median)	90
Lin et al., 2017 [30]	39 (42)	63 (42–82)	Cervix (*n* = 39) Urinary bladder (*n* = 3)	Acute hematuria, dysuria and urgency and frequency of urination	Clinical and cystoscopic findings	Multiplace	2.5	38 (mean)	120
Ribeiro de Oliveira et al., 2015 [31]	108 (176)	61.91 (15–85)	Cervix (*n* = 89) Prostate (*n* = 56) Endometrium (*n* = 17) Bladder (*n* = 7) Rectum (*n* = 3) Ewing’s sarcoma (*n* = 2) Ovarian (*n* = 1) Vulva (*n* = 1)	Cystitis	Resolution of macroscopic hematuria	Multiplace	2.5	36.53 (mean)	90
Mougin et al., 2016 [32]	6 (71)	72 (39–87)	Cervix (*n* = 6) Prostate (*n* = 61) Bladder (*n* = 2) Other (*n* = 2)	Cystitis	CTCAE scale, clinical changes	Multiplace	2.5	29 (mean)	90
Ferreira et al., 2014 [33]	36 (70)	66.5 (34–91)	Cervix (*n* = 34) Vagina (*n* = 2) Prostate (*n* = 30) Anus (*n* = 2) Rectum (*n* = 1) Colon (*n* = 1)	Cystitis, proctitis, enteritis, vaginitis, proctoenteritis	LENT-SOMA scale and clinical	Multiplace	2.4	40 (median)	80
Fink et al., 2006 [34]	14	52.9 (34–77)	Cervix (*n* = 8) Vulvar (*n* = 1) Bartholin gland (*n* = 1) Vaginal (*n* = 1) Ovarian (*n* = 1)	Delayed radiation injuries	Clinical	NR	2.4	32.8 (mean)	90

a: four patients received 2.0 ATA in the hyperbaric chamber b: nine patients received less than 38 treatments c: most of the patients received HBOT for at least 18 times d: some patients received ten extra sessions e: patients had a 5 min air-break at 45 min f: median age

* = calculated age or number of sessions for gynaecological malignancies only

*NR* Not reported

### Technical characteristics regarding HBOT

In Table 1, all information about the technical characteristics of hyperbaric oxygen therapy (HBOT) are reported. HBOT was used in all studies, with pressure varying from 2.0 to 3.0 atmospheric pressure (ATA). The number of sessions varied between 18 and 44. The time of the sessions was ninety minutes by default but could range from sixty to hundred-twenty minutes.

### Time

The time between radiation and injury, the time between injury and therapy and the time to follow-up are reported in Additional file 8.

### Outcome measures

Fifteen [17, 19–26, 29–34] out of twenty-one studies used clinical outcome measures. The Expanded Prostate Index Composite (EPIC) score was used in two studies [14, 16] and can be divided into a urinary and bowel domain. In addition, the Common Terminology Criteria for Adverse Events (CTCAE) scale has been used as a measure of gastrointestinal symptoms [15] and hematuria [32]. The Radiation Therapy Oncology Group (RTOG) criteria is based on the severity of the symptoms [27]. Finally, the LRMGS determines the Late Radiation Morbidity Grading Scheme.

### Patient reported outcome measures

In addition to these outcome measures, the included studies used Patient Reported Outcome Measures (PROMS). The Late Effect Normal Tissues—Subjective Objective Management and Analytic (LENT—SOMA) score reflects the severity of the radiation induced problems and is represented by grades [35]. Jones 2006 [23] has not published or described the LENT-SOMA questionnaire and the questionnaires of the other studies differed. In addition, the 36-item Short Form (SF-36) scale and the Karnofsky score were used as the primary outcome for quality of life [16, 18]. The National Cancer Institute common toxicity criteria (NCI CTC) are related to the toxicity of tissues [22]. For a detailed overview of all outcome measures, see Table 1.

### Difference of reported studies

Of all 21 included studies, 9 evaluated the treatment effects of HBOT on late radiation-induced tissue toxicity (LRITT) in gynaecological malignancies and 12 evaluated the effects of HBOT on LRITT in pelvic malignancies. As in the latter group it was not clearly reported where the primary tumour was located it was not possible to study the specific effects of HBOT on gynaecological malignancies. Therefore, both types of studies evaluating these different study populations are separately discussed below.

### Results studies gynaecological malignancies

Table 2 shows all information about the results in the 9 studies investigating the gynaecological malignancies related to proctitis, cystitis, wound complications and PROMS.

**Table 2:** Results and significance level of gynaecological malignancies

Study, year	Results	Significance level
Oliai et al., 2012 [17]	50% (one out of two patients) time to bleeding recurrence after HBOT was 17 months and a reduction from persistent to intermittent hematuria was reported	NR
	50% (one out of two patients) time to bleeding recurrence after HBOT was 3 months. The patient had a recurrence from persistent to intermittent hematuria, was diagnosed subsequently with bladder cancer after HBOT and underwent 30 extra treatments	
	0.89 mean reduction LENT-SOMA score	
Sidik et al., 2007 [18]	43.41% LENT-SOMA difference between treatment and control group soon after intervention	*p*-value LENT-SOMA soon < 0.001
	13.95% LENT-SOMA difference between treatment and control group six months after HBOT	p-value LENT-SOMA 6 months = 0.008
	15.14% Karnofsky difference between treatment and control group soon after intervention	p-value Karnofsky soon < 0.001
	12.80% Karnofsky difference between treatment and control group six months after HBOT	p-value Karnofsky 6 months = 0.007
Parra et al., 2011 [20]	100% (four out of four patients) complete resolution of macroscopic bleeding after HBOT	NR
Rud et al., 2009 [21]	50% of the patients reported some or good effect	NR
	50% of the patients experienced big changes such as major fractures and/or marked soft tissue oedema	
	Insignificant difference in use or frequency of pain descriptors after HBOT, the use of analgesics, BPI or depression scale scores and MADRS after HBOT	
	MR imaging showed signal abnormalities in 93.75% of the patients and a variety of changes was reported	
Safra et al., 2008 [22]	100% resolution of macroscopic hematuria	
	100% resolution of scar complications	
	3.0 points mean improvement of CTC change in cystitis and proctitis	p-value CTC score = 0.001
	2.8 points mean improvement of CTC change in recto-vaginal fistulas, vesico-vaginal fistulas and vaginal ulcers	
	4.0 points mean improvement of CTC change in wound healing complications	
Williams et al., 1992 [24]	92.9% of the patients had a complete recovery or improvement of necrosis and fistulas	NR
Feldmeier et al., 1996 [25]	61.3% of the patients recovered from the injuries after HBOT	NR
	6.5% of the patients did not recover from the injuries after HBOT	
	25.8% of the patients received inadequate number of treatments and were all deceased	
	6.5% of the patients were lost to follow-up	
Al-Ali et al., 2010 [26]	100% (two out of two patients) reported no response in the treatment group to hemorrhagic cystitis	NR
	100% (one out of one patient) had spontaneous bleeding stop in the control group	
Fink et al., 2006 [34]	71.4% of the patients recovered from delayed radiation injuries or improved more than 50%	NR
	14.3% of the patients (two patients) bleeding recurred after 10 and 11 months	
	Highest success rate in patients with necrotic ulcers with 50% of the patients having complete healing and 50% of the patients achieving a 50% improvement	

*NR* Not reported

#### Cystitis

Oliai 2012 [17], Parra 2011 [20], Safra 2008 [22] and Al-Ali 2010 [26] evaluated patients with cystitis who had suffered from gynaecological malignancies. Oliai 2012 [17] reported a mean reduction of the LENT-SOMA score of 0.89. In addition, Parra 2011 [20] reported a 100% complete resolution of macroscopic bleeding after HBOT. Safra 2008 [22] reported a 100% resolution of macroscopic hematuria and 3.0 points mean improvement of CTC change in cystitis (p = 0.001). Although three out of four studies reported an improvement in cystitis, only Safra 2008 [22] has reported p-values (p = 0.001). Al-Ali 2010 [26] reported no response to HBOT in the treatment group for hemorrhagic cystitis.

#### Proctitis and overall bowel symptoms

Safra 2008 [22] reported improvement in proctitis after HBOT. This study reported a significant 3.0 points mean improvement of CTC change in proctitis (*p* = 0.001) [22].

#### Wound complications

Safra 2008 [22], Williams 1992 [24], Feldmeier 1996 [25] and Fink 2006 [34] reported improvement in wound complications. Safra 2008 [22] reported 100% resolution of scar complications and 4.0 points mean improvement of the CTC change in wound healing complications (*p* = 0.001). In addition, Williams 1992 [24] reported a complete recovery or improvement of necrosis and fistulas in 92.9% of the patients. Feldmeier 1996 [25] reported a recovery from the injuries after HBOT in 61.3% of the patients. Finally, Fink 2006 [34] reported a complete healing of necrotic ulcers in 50% of the patients and a 50% improvement in 50% of the patients. Only Safra 2008 [22] reported a significant *p*-value (*p* = 0.001).

#### Patient reported outcome measures

Multiple studies have published PROMS, including quality of life, pain and depression symptoms. Sidik 2007 [18] reported a significant difference of 13.95% between the control group and the treatment group in the LENT-SOMA score (*p* = 0.008) and a difference of 12.80% in the Karnofsky score (*p* = 0.007) six months after HBOT. Rud 2009 [21] reported no improvement in the Brief Pain Inventory (BPI) score or Montgomery and Asberg Depression Rating Scale (MADRS) but fifty percent of the patients noticed some or good effect after treatment. This study did not report p-values at all.

### Results studies pelvic radiotherapy

Twelve of the twenty-one studies included patients with LRITT after pelvic radiotherapy. For a detailed overview of these studies, see Table 3.

**Table 3:** Results and significance level of pelvic radiotherapy

Study, year	Percentage of gynaecological cancers	Results	Significance level
Oscarsson et al., 2013 [14]	5.1%	Relative improvement of the EPIC score urinary domain immediately after treatment was 22% and after six to twelve months follow-up the relative improvement was 21%	*p*-value EPIC urinary score relative increase < 0.001
		29% improvement EPIC score urinary domain in early state and after six to twelve months follow-up in patients with an EPIC score below eighty. In the patients with an EPIC score below eighty before HBOT, 76% of the patients improved after HBOT and 24% did not respond to HBOT	Improved EPIC score p-value < 0.001
		31% of the patients reported an EPIC score above eighty after HBOT in the urinary domain	
		Relative improvement EPIC score bowel domain immediately after treatment was 24% and after six to twelve months follow-up was 21%	*p*-value EPIC bowel score relative increase < 0.001
		41% increasement of EPIC score bowel domain early after HBOT and 39% increasement of EPIC score bowel domain six to twelve months after HBOT in patients with an EPIC score below eighty	Improved EPIC score *p*-value < 0.001
		89% of patients had an increase in EPIC score after HBOT and 11% of patients did not respond to HBOT in the patients with an EPIC score below eighty	
		22% of the patients reported an EPIC score above eighty in the bowel domain	
Glover et al., 2016 [15]	45.2%	Absolute difference between treatment group and control group in improvement of at least one point in IBDQ rectal bleeding score after twelve months: 7.6%	*p*-value absolute difference IBDQ-score = 0.58 95% CI = −20.3 to 35.5
		Insignificant difference in improvement of overall bowel function after twelve months between treatment and control group (Mann–Whitney U-score: 0.67)	U-score bowel function p-value = 0.50
		Insignificant difference in rectal bleeding after twelve months between treatment and control group (Mann-Whitney U-score: 1.69)	U-score rectal bleeding p-value = 0.092
		The improvement from baseline to twelve months was consistent with the ITT analysis and differed with a U score of 0.71 for overall bowel function and a U score of 2.06 for rectal bleeding between the control and treatment group	U-score bowel function ITT p-value = 0.48 U-score rectal bleeding ITT p-value = 0.040
		PP-analyses were consistent with the ITT analysis with a U score of 0.94 for overall bowel function and a U score of 1.44 for rectal bleeding	U-score bowel function PP p-value = 0.35 U-score rectal bleeding PP p-value = 0.15
		LENT-SOMA rectal bleeding score: 100% of the patients increased in the control group and 31% of the patients increased in the treatment group	
		Insignificant improvement in rectum and intestine LENT-SOMA score in the control and treatment group. The U-score of rectal LENT-SOMA was 1.62 and the U-score of intestinal LENT-SOMA was -1.41	*p*-value U-score rectal = 0.11 *p*-value U-score intestinal = 0.16
		No difference between treatment and control group in CTCAE grades after treatment	
		Subgroup analyses reported that the IBDQ scores between the treatment and control group did not change in patients who had completed radiotherapy one to five years before HBOT	
		The U score for overall bowel function was 0.59 and the U score for rectal bleeding was 1.57	*p*-value bowel function = 0.56 *p*-value rectal bleeding = 0.12
		Difference in rectal bleeding was reported in the subgroup analyses of patients treated in a monoplace chamber with a U score of 2.9. The difference for overall bowel function in the subgroup analyses of patients treated in a monoplace chamber was insignificant with a bowel function U score of -0.31	p-value rectal bleeding monoplace = 0.004 *p*-value bowel function monoplace = 0.76
Oscarsson et al., 2019 [16]	25.3%	73% of the patients had an improvement, 23% of the patients did not change and 5% decreased in the treatment group of the EPIC total urinary score	
		34% of the patients had an improvement, 54% of the patients did not change and 11% decreased in the control group of the EPIC total urinary score	
		40% of the patients in the treatment group and 9% of the patients in control group reported an EPIC score above eighty at the end of the study	
		64% of the patients had an improvement, 28% of the patients did not change and 8% had decreased LRMGS grades in the treatment group	*p*-value differences between groups = 0.0012
		18% of the patients had an improvement, 53% of the patients did not change and 29% had decreased LRMGS grades in the control group	
		10.1 points significant difference in mean EPIC urinary total score between treatment and control group (ITT analysis)	95% CI = 2.2–18.1 ITT analysis p-value = 0.013
		11.4 points significant difference mean EPIC urinary total score between treatment and control group in urinary domain (PP analysis)	95% CI = 3.5–19.2 PP analysis *p*-value = 0.0047
		8.33 points significant difference mean EPIC bowel total score between treatment and control group	95% CI = 1.15—15.54 *p*-value = 0.024
		11.5 points significant difference in the EPIC sub score of urinary bother between treatment and control group	95% CI = 2.7—20.3 *p*-value = 0.012
		12.1 points significant difference in the EPIC sub score of urinary incontinence between treatment and control group	95% CI = 4.3—19.9 *p*-value = 0.0031
		Significant improvement in mean SF-36 score for general health in the treatment group of 13.2 points.	95% CI SF-36 score = 6.0—20.4 *p*-value = 0.0006
		No significant change in the control group for the mean SF-36 score	
Clarke et al., 2008 [19]	86.7%	88.9% of the patients recovered or experienced some improvement in the treatment group and 62.5% experienced some improvement in the control group. The calculated absolute difference was 26.4%	
		The treatment group reported significantly greater healing/improvement compared to the control group	Fisher’s exact test *p* = 0.0009 Logistic regression analysis *p* = 0.0011
		2.39 points absolute difference in improvement of the LENT-SOMA score between treatment and control group. Improvement in treatment group was greater than in the control group	*p*-value absolute difference < 0.0001 greater decrease p-value = 0.0019
		Treatment group had a lower mean score than the control group after initial allocation with a difference of 1.93	95% CI = 0.38–3.48 *p*-value difference in mean score = 0.0150
		No differences were reported after the crossover	*p*-value after crossover = 0.6594
		Odds ratio for some improvement was 5.93	95% CI = 2.04–17.24
		Significant better outcomes were reported more often in the treatment group An absolute risk reduction of 0.32 (32%) was recorded in the clinical evaluation outcomes, which corresponds to a number needed to treat of 3 Improvement in treatment group for bowel bother was 14% and for bowel function 9%. Improvement in control group for bowel bother was 5% and for bowel function 6% The control group had an improvement of 13.6 for bowel bother and 10% for bowel function after cross-over	*p*-value Jockheere Terpstra = 0.0008
		A significant improvement was reported between initialization and randomization in the treatment group for the bowel bother subscale with a change of 14.14	*p*-value = 0.0007
		The control group had an insignificant improvement between initialization and randomization for the bowel bother subscale with a change of 5.75	*p*-value = 0.1521
		The control group had a significant improvement after crossover with a change of 14.27	*p*-value = 0.0002
Jones et al., 2006 [23]	60.0%	44.5% complete recovery of rectal bleeding, 33.3% decrease in frequency and severity of rectal bleeding 11.1% of the patients had a decrease in the rectal bleeding 60.0% rectal pain recovery and 20.0% of the patients reported an improvement in rectal pain 20.0% of the patients reported full recovery of diarrhea and 60.0% reported an improvement	NR
Bui et al., 2004 [27]	NR	100% overall improvement	NR
Andren et al., 2020 [28]	13.5%	Significant mean LENT-SOMA score reduction for all patients of 3.7	95% CI mean reduction = 2.6–4.8 *p*-value mean reduction < 0.001
		Significant mean LENT-SOMA score reduction in the subgroup analysis for proctitis of 3.8	95% CI reduction proctitis = 1.4–6.10 *p*-value reduction proctitis = 0.004
		Significant mean LENT-SOMA score reduction in the subgroup analysis for cystitis of 3.7 (2.4–5.0)	*p*-value reduction cystitis < 0.001
		Significant association between severity of LRITT and improvement in LENT-SOMA scores	*p*-value severity of LRITT = 0.003
		Insignificant association between improvement of LENT-SOMA scores and the number of treatments, number of comorbidities and age	*p*-value number of treatments = 0.71 *p*-value number of comorbidities = 0.50 *p*-value of age = 0.21
Ngoo et al., 2018 [29]	NR	The bleeding resolved in 77.8% of the patients	
		This percentage was associated with a shorter time between radiotherapy and the first cystitis episode and was associated with lower transfusion requirements before treatment	*p*-value interval = 0.018 *p*-value lower transfusion requirements = 0.012
Lin et al., 2017 [30]	92.9%	Macroscopic hematuria resolved in 83.3% of the patients after an average of 38 sessions and macroscopic hematuria decreased in 7.1% of the patients	NR
		Three patients (7.1%) had frequent urination and urgency without significant hematuria, with symptoms resolved after HBOT	
		One patient (2.4%) did not respond to HBOT	
		One patient underwent an urodynamic test with the following results: urine peak flow from 12.8 ml/s before HBOT to 15.0 ml/s after HBOT, urine mean flow from 6.5 ml/s before HBOT to 8.9 ml/s after HBOT, urine voiding time of 40.0 s before HBOT to 28.0 s after HBOT, time to peak flow from 15.0 s before HBOT to 8.0 s after HBOT and voided volume from 251 mL before HBOT to 248 mL after HBOT	
Ribeiro de Oliveira et al., 2015 [31]	61.4%	67% of the patients recovered completely from hematuria and 22.7% of the patients recovered partially from hematuria 10.2% of the patients did not recover from hematuria of which 9.1% of these patients had an absence of variation of hematuria and 1.1% of these patients had aggravation of hematuria After a mean follow-up period of twelve months, the recurrence rate of hematuria was 15.2%	
		No significant difference of hematuria resolution between sex groups	*p*-value = 0.738
		No significant difference in hematuria resolution between uterine cervix cancer patients and prostate cancer patients	*p*-value = 0.228
		Significant difference for the need of transfusion support in the group with hematuria resolution and the group without hematuria resolution. 82.9% of the patients in the group with hematuria resolution did not use transfusion therapy and 61.1% of the patients in the group without hematuria resolution did not use transfusion therapy	*p*-value = 0.026
		Insignificant difference in hematuria resolution depending on the differences in time between radiotherapy and hematuria, time between hematuria and HBOT and time between radiotherapy and HBOT	*p*-value radiotherapy and hematuria = 0.236 p-value hematuria and HBOT = 0.199 *p*-value radiotherapy and HBOT = 0.44
		Significant difference in hematuria resolution depending on the number of treatments	*p*-value number of treatments = 0.042
Mougin et al., 2016 [32]	8.5%	Haematuria had completely resolved in 52.1% of the patients In 12.7% of the patients, haematuria had partially resolved No improvement was registered in 35.2% of the patients 26.8% of the patients had a recurrence of haematuria after a median follow-up of 15 months, of which 9 patients received a second HBOT course that helped 8 patients At 1 year, the haematuria-free survival rate was 70%	
		The hematuria grade of less than 3 made a significant difference for successful therapeutic outcome with a hazard ratio of 4.4 (univariate analysis)	*p*-value = 0.01
		The hematuria grade of less than 3 at the time of diagnosis made a significant difference for successful therapeutic outcome with a hazard ratio of 3.6 (multivariate analysis)	*p*-value = 0.027
		The anticoagulant therapy made a significant difference for treatment failure with a hazard ratio of 0.3	*p*-value = 0.03
Ferreira et al., 2014 [33]	51.4%	The response rate of resolution or improvement of haematuria after a median follow-up period of 55.5 months was 91.4%	
		Haematuria persisted in 6 patients, of which 5 patients had undergone cystectomy	
		Median difference in subjective score of dysuria before and after HBOT was 1 (1–1.5)	*p*-value < 0.001
		Median difference in subjective score of frequency before and after HBOT was 0.5 (0.5–1.5)	*p*-value = 0.016
		Median difference in subjective score of haematuria before and after HBOT was 2.5 (2–2.5)	*p*-value < 0.001
		Median difference in subjective score of incontinence before and after HBOT was 0.5 (0–1)	*p*-value = 0.003
		Median difference in subjective score of decreased stream before and after HBOT was 0 (0–1)	*p*-value = 0.14
		The median difference in sum of all subjective scores before and after HBOT was 5 (5–6)	*p*-value < 0.001
		Significant difference was reported between haematuria response and the time interval between the first episode of haematuria and HBOT	*p*-value < 0.05

NR = not reported

#### Cystitis

Oscarsson 2013 [14], Oscarsson 2019 [16], Andren 2020 [28], Ngoo 2018 [29], Lin 2017 [30], Ribeiro de Oliveira 2015 [31], Mougin 2016 [32] and Ferreira 2014 [33] evaluated patients with cystitis who had suffered from pelvic malignancies. All studies reported an improvement in cystitis symptoms. Oscarsson 2013 [14] reported a significant improvement in the EPIC score in the urinary domain (*p* < 0.001). Secondly, Oscarsson 2019 [16] reported a significant improvement in the EPIC score (*p* = 0.013 and *p* = 0.0047) and LRMGS grades (*p* = 0.0012). In addition, Andren 2020 [28] reported a significant mean LENT-SOMA score reduction for cystitis of 3.7 (*p* < 0.001). Ngoo 2018 [29] reported a bleeding resolution in 77.8% of the patients and Lin 2017 [30] reported a resolution of macroscopic haematuria in 83.3% of the patients and a decrease of macroscopic haematuria in 7.1% of the patients. Moreover, Ribeiro de Oliveira 2015 [31] reported complete recovery from haematuria in 67% of the patients and a partially recovery from haematuria in 22.7% of the patients. Mougin 2016 [32] reported a complete resolution of haematuria in 52.1% of the patients and a partially resolution of haematuria in 12.7% of the patients. Finally, Ferreira 2014 [33] reported a response rate of haematuria resolution or haematuria improvement after a median follow-up period of 55.5 months of 91.4%. Ferreira 2014 [33] also reported a significant median difference in the sum of subjective LENT-SOMA scores before and after HBOT of 5 (*p* < 0.001). All studies except Lin 2017 [30] reported p-values. Ngoo 2018 [29], Ribeiro de Oliveira 2015 [31] and Mougin 2016 [32] only reported p-values of the results in subgroup analyses.

#### Proctitis and overall bowel symptoms

Glover 2016 [15] reported insignificant improvement in the Mann–Whitney U score (*p* = 0.50 and *p* = 0.092) and the Inflammatory Bowel Disease Questionnaire (IBDQ) rectal bleeding score (*p* = 0.12). This study also reported an insignificant improvement in the LENT-SOMA score (*p* = 0.11 and *p* = 0.16) and no differences in the CTCAE grades were found. Moreover, Oscarsson 2013 [14] and Oscarsson 2019 [16] reported a significant improvement in the EPIC score in the bowel domain with respectively a *p*-value < 0.001 and a 95% CI of 1.15 to 15.54. Clarke 2008 [19] reported a significant better improvement in the treatment group for the LENT-SOMA score with a difference of 2.39 points (*p* < 0.0001), a significant greater healing or improvement in the treatment group with a difference of 26.4% and a 32% risk reduction was found. Andren 2020 [28] reported a significant mean LENT-SOMA score reduction for proctitis of 3.8 (*p* = 0.004). Finally, Jones 2006 [23] reported an improvement in proctitis complaints, with no p-values reported.

#### Patient reported outcome measures

Bui 2004 [27] reported a 100% overall improvement in late side effects of pelvic radiotherapy, but no p-values have been reported. In addition, Oscarsson 2019 [16] reported a significant improvement in the mean SF-36 score for general health in the treatment group of 13.2 points (*p* = 0.0006).

### Comparison of the symptoms

Within the included patient group, gynaecological cancer patients with wound complications benefit most from HBOT compared to other late side effects of LRITT with a range of 50%-100% resolution in three of the four studies. All four studies reported a marked improvement in patients with wound complications after HBOT.

### Side effects

Fifteen studies [15–17, 19, 20, 23–25, 27–29, 31–34] reported adverse effects after HBOT and six studies [14, 18, 21, 22, 26, 30] did not report adverse effects after HBOT. In this study, the most reported adverse effects of HBOT were barotraumas or other complications in the ears, which is reported in fourteen studies. The calculated incidence of this symptom reported in these studies is approximately 1:10 [15–17, 19, 20, 23, 24, 27–29, 31–34]. In this review, the calculated percentage of patients with barotrauma or other complications in the ears was 10.3% and approximately corresponded to the study Blanshard 1996 [36]. A second common side effect reported in the included studies was myopia, or other complications in the eyes, with a calculated incidence of approximately 1:25 [15, 16, 19, 27, 28, 32]. A detailed overview of the side effects is presented in Additional file 9.

## Discussion

This review demonstrated that HBOT is an effective and safe way to treat LRITT in women with treated gynaecological cancers in reported complaints of proctitis, cystitis, wound complications and Patient Reported Outcome Measures (PROMS). All but three studies [15, 21, 26] investigating LRITT reported a positive result after the HBOT. In addition, nine studies [14, 16, 18, 19, 22, 28, 29, 32, 33] reported significant p-values. A low incidence of adverse effects after HBOT has been reported.

Several studies had been published regarding the treatment effects of HBOT in patients with LRITT. Most studies conclude that HBOT has a positive effect on gynaecological patients with LRITT. This review is therefore consistent with previous systematic reviews such as Craighead 2011 [37] and Allen 2012 [11]. This systematic review specifically investigated the effect of HBOT on various symptoms of LRITT in patients with treated gynaecological malignancies, whereas previous studies have often examined one symptom such as cystitis only or proctitis only.

All studies except Al-Ali 2010 [26] reported positive results with regard to HBOT on cystitis complaints [17, 20, 22]. However, only one study [22] reported significant p-values which indicates a low validity. Four studies [14, 16, 28, 33] investigating the effect of HBOT on cystitis in pelvic radiotherapy, showed a significant positive effect of HBOT and two studies [29, 32] reported significant p-values in the subgroup analyses. Most studies have reported a positive effect, so patients with cystitis may benefit from HBOT.

Regarding proctitis as LRITT, all seven studies except Glover 2016 [15] reported positive effects. These studies focused either on gynaecological malignancies or on patients with pelvic radiation. From this it is demonstrated that patients with proctitis may benefit from HBOT.

All four studies that examined wound complications in the vaginal, vulvar and rectovaginal area in gynaecological malignancies reported a positive result. The range for complete resolution of scar complications, necrotic ulcers and the healing of injuries was 50%-100% in three studies [22, 25, 34]. However, only one study [22] reported a significant result. From these results it can be concluded that gynaecological patients with wound complications in these areas benefit most from HBOT compared to other late side effects of LRITT.

Finally, three of the four studies [16, 18, 27] investigating Patient Reported Outcome Measures (PROMS), including quality of life, pain and depression symptoms reported positive results in gynaecological malignancies and pelvic radiotherapy. Sidik 2007 [18] showed a significant difference between the control and treatment group, Bui 2004 [27] reported a hundred percent overall improvement and Oscarsson 2019 [16] reported an improvement in mean SF-36 score for general health of 13.2 points. Therefore, it can be concluded that in most patients the quality of life, pain and depression symptoms improved after HBOT.

The main strength of this review was the fact that different symptoms of LRITT were compared with each other, which has not been done in previous studies.

However, an important limitation of this review is the low quality of the included studies. Although there appears to be a benefit in treating gynaecological cancer patients suffering from LRITT with HBOT, few studies have reported significance, studies included few patients, studies published descriptive results, and different outcome measures were used in the included studies, making the studies difficult to compare. Because of this low quality of the included studies and the difficulty to compare the studies, the conclusion that HBOT offers a benefit in gynaecological cancer patients who suffer from LRITT should be taken with care.

Only Ferreira 2014 [33] reported patients experiencing vaginitis. The effect of HBOT on sexual disfunction due to radiotherapy will require further investigation in future studies, as pelvic radiotherapy plays a significant negative role in sexual dysfunction [38]. Ideally, more high-quality studies should be done to be included in a future review in order to improve the reliability of the study results. Moreover, the long-term effect could be evaluated in future research, as most included studies reported a follow-up period of two years or less.

## Conclusion

From this review it can be concluded that the hyperbaric oxygen therapy (HBOT) has a positive effect on late radiation-induced tissue toxicity (LRITT) in gynaecological malignancies. Within the included patient group, gynaecological patients with wound complications localized in the vaginal, vulvar and rectovaginal area benefit most from this treatment compared to other late side effects of LRITT. The hyperbaric oxygen therapy can therefore be used in women who suffer from LRITT three months after their radiation for gynaecological cancers has ended. However, an important limitation of this review is the low quality of the included studies.

Future studies should be of higher quality in order to improve the reliability of obtained evidence so far. Moreover, the long-term effect of HBOT on LRITT in treated gynaecological malignancies should be investigated in subsequent studies. Future studies should also investigate the effect of HBOT on sexual disfunction in treated gynaecological cancer patients. Finally, future studies could further investigate the effect of HBOT on the quality of life in treated gynaecological cancer patients by using the European Organization for Research on Treatment of Cancer (EORTC) questionnaires.

## Supplementary Information

**Additional file 1. Table 4**. Literature search in PubMed.

**Additional file 2. Table 5**. Literature search in Embase.

**Additional file 3. Table 6**. Literature search in the Cochrane Library.

**Additional file 4. Table 7**. Reasons of exclusion from PubMed search.

**Additional file 5. Table 8**. Reasons of exclusion from Embase search.

**Additional file 6. Table 9**. Reasons of exclusion from the Cochrane library search.

**Additional file 7. Table 10**. Study design of the included studies.

**Additional file 8. Table 11**. Time.

**Additional file 9. Table 12**. Reported side effects.

## Data Availability

All data generated or analysed during this study are included in this published manuscript.

## Abbreviations

HBOT: Hyperbaric oxygen therapy
LRITT: Late radiation-induced tissue toxicity
PROMS: Patient reported outcome measures
ATA: Atmospheric pressure
ROS: Reactive oxygen species
RNS: Reactive nitrogen species
SPc: Stromal progenitor cells
LENT-SOMA: Late effects normal tissue task force–subjective, objective, management, analytic
EPIC: Expanded prostate index composite
IBDQ: Inflammatory bowel disease questionnaire
CI: Confidence interval
SD: Standard deviation
OR: Odds ratio
CTCAE: Common terminology criteria for adverse events
RTOG: Radiation therapy oncology group
LRMGS: Late radiation morbidity grading scheme
SF-36: 36-Item short form
NCI CTC: National cancer institute common toxicity criteria
BPI: Brief pain inventory
MADRS: Montgomery and asberg depression scale
EORTC: European organization for research on treatment of cancer
