# Supplementary material for: Hyperbaric oxygen treatment for late radiation-induced tissue toxicity in treated gynaecological cancer patients: a systematic review
Source: Radiat Oncol. 2022 Oct 6;17:164. doi: 10.1186/s13014-022-02067-6 (PMC9540739; doi:10.1186/s13014-022-02067-6)
Supplement: Supplementary file 1 — Additional file 1. Table 4. Literature search in PubMed. [file 13014_2022_2067_MOESM1_ESM.pdf]

Table 4. Literature search in PubMed

Main Term	Search	Items found
Gynaecologic cancer types	((((((((((((((((((((((((((((((((((((uterus[Title]) OR uterus[MeSH Terms]) OR womb[Title/Abstract]) OR womb[MeSH Terms]) OR matrix[Title/Abstract]) OR matrix[MeSH Terms]) OR myometrium[Title/Abstract]) OR myometrium[MeSH Terms]) OR cervix[Title/Abstract]) OR cervix[MeSH Terms]) OR uterine cervix[Title/Abstract]) OR uterine cervix[MeSH Terms]) OR cervix uteri[Title/Abstract]) OR cervix uteri[MeSH Terms]) OR ovary[Title/Abstract]) OR ovary[MeSH Terms]) OR ovaries[Title/Abstract]) OR ovaries[MeSH Terms]) OR ovaries[Title/Abstract]) OR ovarian[MeSH Terms]) OR ovarian[Title/Abstract]) OR labia minora[MeSH Terms]) OR labia minora[Title/Abstract]) OR labia majora[MeSH Terms]) OR labia majora[Title/Abstract]) OR lip[MeSH Terms]) OR lip[Title/Abstract]) OR lips[MeSH Terms]) OR lips[Title/Abstract]) OR rims[MeSH Terms]) OR rims[Title/Abstract]) OR brims[MeSH Terms]) OR brims[Title/Abstract]) OR overlaps[MeSH Terms]) OR overlaps[Title/Abstract]) OR vagina[MeSH Terms]) OR vagina[Title/Abstract] OR vulvar[MeSH Terms] OR vulvar[Title/Abstract]	**892785**
	**OR**	
Gynecologic	(((((((gynecol[Title/Abstract]) OR gynecol[MeSH Terms]) OR gynecology[Title/Abstract]) OR gynecology[MeSH Terms]) OR gynaecological[Title/Abstract]) OR gynaecological[MeSH Terms]) OR obstetrics[Title/Abstract]) OR obstetrics[MeSH Terms]	**73337**
	**AND**	
Hyperbaric oxygen therapy	(((((((((((hyperbaric oxygen treatment[Title/Abstract]) OR hyperbaric oxygen treatment[MeSH Terms]) OR hyperbaric oxygen treatments[Title/Abstract]) OR hyperbaric oxygen treatments[MeSH Terms]) OR hyperbaric oxygenation[Title/Abstract]) OR hyperbaric oxygenation[MeSH Terms]) OR hyperbaric oxygenations[Title/Abstract]) OR hyperbaric oxygenations[MeSH Terms]) OR hyperbaric oxygen therapy[Title/Abstract]) OR hyperbaric oxygen therapy[MeSH Terms]) OR hyperbaric oxygen therapies[Title/Abstract]) OR hyperbaric oxygen therapies[MeSH Terms]	**13509**
	**Combined total**	**226**
