# Supplementary material for: Hyperbaric oxygen treatment for late radiation-induced tissue toxicity in treated gynaecological cancer patients: a systematic review
Source: Radiat Oncol. 2022 Oct 6;17:164. doi: 10.1186/s13014-022-02067-6 (PMC9540739; doi:10.1186/s13014-022-02067-6)
Supplement: Supplementary file 2 — Additional file 2. Table 5. Literature search in Embase. [file 13014_2022_2067_MOESM2_ESM.pdf]

Table 5. Literature search in Embase

Search	Items found
('gynecological cancer'/exp OR 'gynecological cancer' OR (gynecological AND ('cancer'/exp OR cancer)) OR 'female genital tract cancer'/exp OR 'female genital tract cancer') AND ('hyperbaric oxygen therapy'/exp OR 'hyperbaric oxygen therapy')	**173**
