# Supplementary material for: Hyperbaric oxygen treatment for late radiation-induced tissue toxicity in treated gynaecological cancer patients: a systematic review
Source: Radiat Oncol. 2022 Oct 6;17:164. doi: 10.1186/s13014-022-02067-6 (PMC9540739; doi:10.1186/s13014-022-02067-6)
Supplement: Supplementary file 3 — Additional file 3. Table 6. Literature search in the Cochrane Library. [file 13014_2022_2067_MOESM3_ESM.pdf]

Table 6. Literature search in the Cochrane Library

Search	Items found
MeSH Descriptor: [Hyperbaric Oxygenation] explode all trees OR {''hyperbaric oxygen therapy''):ti,ab,kw AND MeSH descriptor: [Genital Diseases, Female] explode all trees OR (gynecological cancer):ti,ab,kw	**18**
