# Supplementary material for: Hyperbaric oxygen treatment for late radiation-induced tissue toxicity in treated gynaecological cancer patients: a systematic review
Source: Radiat Oncol. 2022 Oct 6;17:164. doi: 10.1186/s13014-022-02067-6 (PMC9540739; doi:10.1186/s13014-022-02067-6)
Supplement: Supplementary file 4 — Additional file 4. Table 7. Reasons of exclusion from PubMed search. [file 13014_2022_2067_MOESM4_ESM.pdf]

Table 7. Reasons of exclusion from PubMed search

Author(s)	Year	Journal abbreviation	Reason of exclusion
Watson et al	1970	Br J Radiol.	Article not available
Roulston et al	1968	J Obstet Gynaecol Br Commonw.	Article not available
Johnson et al	1967	Natl Cancer Inst Monogr.	Article not available
Proshina et al	1985	Anesteziol Reanimatol.	Article not available
Ward et al	1979	Br J Radiol.	Article not available
Wakabayashi et al	1971	Nihon Igaku Hoshasen Gakkai Zasshi.	Article not available
Huddy et al	2006	Eur J Gastoenterol Hepatol.	Case report
Harlan et al	2015	Undersea Hyperb Med.	Case report
Kärcher et al	1975	Wien Klin Wochenschr.	Case report
Dische et al	1972	Br J Radiol.	Case report
King et al	2012	Arch Otolaryngol Head Neck Surg.	Case report
Yazawa et al	1995	Int Utol Nephrol.	Case report
Liu et al	2017	Medicine (Baltimore).	Case report
Bruni et al	1972	J Oral Surg.	Case report
Maurel et al	2020	Gynecol Obstet Fertil Senol.	Case report
Wang et al	1999	Eur J Obstet Gynecol Reprod Biol.	Case report
Abu-Asi et al	2013	Support Care Cancer.	Case series
Nakada et al	1992	Eur Urol.	Case series
Griffiths et al	2018	Gynecol Oncol Rep.	Case series
Da Silva et al	2018	Childs Nerv Syst.	Exclusive patient group
Nie et al	2014	Sheng Li Xue Bao.	Exclusive patient group
You et al	2014	Undersea Hyperb Med.	Exclusive patient group
Meah et al	2018	J Low Genit Tract Dis.	Exclusive patient group

Hu et al	2017	Stroke.	Exclusive patient group
Limirio et al	2018	PLoS One.	Exclusive patient group
Dai et al	2015	Ann Plast Surg.	Exclusive patient group
Chen et al	2014	Neuroscience.	Exclusive patient group
Soejima et al	2013	Exp Neurol.	Exclusive patient group
Hayashi et al	2012	J Reprod Dev.	Exclusive patient group
Frezza et al	2015	Acta Trop.	Exclusive patient group
Bilici et al	2015	Facial Plast Surg.	Exclusive patient group
Yalcin et al	2014	Undersea Hyperb Med.	Exclusive patient group
Zhao et al	2011	Exp Brain Res.	Exclusive patient group
Hsu et al	2004	J trauma.	Exclusive patient group
Zhang et al	2005	Zhonghua Gan Zang Bing Za Zhi.	Exclusive patient group
Vlodavsky et al	2006	Neuropathol Appl Neurobiol.	Exclusive patient group
Veltkamp et al	2006	Brain Res.	Exclusive patient group
Atis et al	2012	Reprod Biol Endocrinol.	Exclusive patient group
Ishii et al	2002	J Orthop Res.	Exclusive patient group
Syftestad et al	1976	Undersea Biomed Res.	Exclusive patient group
Lammers et al	2008	Am J Physiol Heart Circ Physiol.	Exclusive patient group
Muhonen et al	2006	Int J Oral Maxillofac Surg.	Exclusive patient group
Nguyen et al	2020	Wound Repair Regen.	Exclusive patient group
Rattazzi et al	1980	Birth Defects Orig Artic Ser.	Exclusive patient group
Pires et al	2002	Prog Urol.	Language
Bates et al	1974	Br J Radiol.	Letter
Placer et al	1990	Enferm Infecc Microbiol Clin.	Letter
Corcoran et al	2017	Prostaglandines Leukot Essent Fatty Acids.	No gynaecological malignancies

Tao et al	2019	Exp Ther Med.	No gynaecological malignancies
Tlapák et al	2020	Undersea Hyperb Med.	No gynaecological malignancies
Jean dit Gautier et al	2015	Int J Gynaecol Obstet.	No gynaecological malignancies
Condemi et al	2018	Eur Rev Med Pharmacol Sci.	No gynaecological malignancies
Al Hadi et al	2015	J Dent.	No gynaecological malignancies
Michalski et al	2011	Acta Neurol Scand.	No gynaecological malignancies
Zhang et al	2013	J Invest Dermatol.	No gynaecological malignancies
Costa et al	2015	Acta Med Port.	No gynaecological malignancies
Jacomini et al	2012	Undersea Hyperb Med.	No gynaecological malignancies
Cherng et al	2012	Ann Plast Surg.	No gynaecological malignancies
Yang et al	2013	Life Sci.	No gynaecological malignancies
Douso et al	2009	J Minim Invasive Gynecol.	No gynaecological malignancies
Jung et al	2010	Head Face Med.	No gynaecological malignancies
Svensson et al	2008	J Pediatr Surg.	No gynaecological malignancies
Shyu et al	2009	Clin Sci (Lond).	No gynaecological malignancies
Ostrowski et al	2010	Acta Neurochir Suppl.	No gynaecological malignancies
Lekic et al	2011	Acta Neurochir Suppl.	No gynaecological malignancies
Alleva et al	2008	Mol Med.	No gynaecological malignancies
Romero-Valdovinos et al	2011	In Vitro Cell Dev Biol Anim.	No gynaecological malignancies
Gordillo et al	2003	Am J Surg.	No gynaecological malignancies
Tompach et al	1997	Int J Oral Maxillofac Surg.	No gynaecological malignancies
Kirschbaum et al	1969	Pediatr Res.	No gynaecological malignancies
Bonito et al	1968	Riv Ital Ginecol.	No gynaecological malignancies
Roberts et al	1994	Br J Dermatol.	No gynaecological malignancies
Myers et al	1990	NCI Monogr.	No gynaecological malignancies

Guillard et al	2010	Undersea Hyperb Med.	No gynaecological malignancies
Wu et al	2010	J Am Podiatr Med Assoc.	No gynaecological malignancies
Farmer et al	1978	Ann Otol Rhinol Laryngol.	No gynaecological malignancies
Hanson et al	1966	Postgrad Med J.	No gynaecological malignancies
Mikhalenko et al	1972	Pediatr Akus Ginekol.	No gynaecological malignancies
Sun et al	2010	J Cereb Blood Flow Metab.	No gynaecological malignancies
Macdougall et al	1967	Exp Cell Res.	No gynaecological malignancies
Chessex et al	2010	Free Radic Biol Med.	No gynaecological malignancies
Rivenzon et al	1965	Oncologia.	No gynaecological malignancies
Thibault et al	2013	Brachytherapy.	No gynaecological malignancies
White et al	2018	J Pediatr Urol.	No gynaecological malignancies
Bakker et al	2000	Ned Tijdschr Geneeskd.	No gynaecological malignancies
Fearmonti et al	2016	Wounds.	No gynaecological malignancies
Babenerd et al	1969	Gynakol Rundsch.	No gynaecological malignancies
Tkachenko et al	1988	Vestn Khir Im I I Grek.	No gynaecological malignancies
Delanian et al	2008	Chir Main.	No gynaecological malignancies
Chaika et al	1990	Akush Ginekol (Mosk).	No gynaecological malignancies
Carl et al	1998	J Prosthet Dent.	No gynaecological malignancies
Asribekova et al	1991	Probl Endokrinol (Mosk).	No gynaecological malignancies
Wakabayashi et al	1970	Gan No Rinsho.	No gynaecological malignancies
Bogdán et al	2005	Orv Hetil.	No gynaecological malignancies
Proshina et al	1982	Med Sestra.	No gynaecological malignancies
Persianinov et al	1978	Anesteziol Reanimatol.	No gynaecological malignancies
Chernukha et al	1986	Akush Ginekol (Mosk).	No gynaecological malignancies
Willis et al	1984	Scand J gastroenterol Suppl.	No gynaecological malignancies

Niezgoda et al	2005	Adv Skin Wound Care.	No gynaecological malignancies
Hong et al	2019	Arch Craniofac Surg.	No gynaecological malignancies
Colón et al	2013	Bol Asoc Med P R.	No gynaecological malignancies
Sidel'nikova et al	1991	Akush Ginekol (Mosk).	No gynaecological malignancies
Burler et al	1989	Akush Ginekol (Mosk).	No gynaecological malignancies
Nigro et al	2018	Plast Reconstr Surg Glob Open.	No gynaecological malignancies
Yang et al	2017	Med Gas Res.	No gynaecological malignancies
Asadamongkol et al	2014	Med Gas Res.	No gynaecological malignancies
Hart et al	2012	Adv Wound Care (New Rochelle).	No gynaecological malignancies
Mrdjenovich et al	2010	J Am Col Cerif Wound Spec.	No gynaecological malignancies
Eggert et al	2007	Wounds.	No gynaecological malignancies
Roberts et al	1994	J Wound Care.	No gynaecological malignancies
Frerich et al	2000	Mund Kiefer Gesichtschir.	No gynaecological malignancies
Arrosagaray et al	1987	J Clin Microbiol.	No gynaecological malignancies
Wu et al	2010	J Vasc Surg	No gynaecological malignancies
Gajendrareddy et al	2017	J Periodontal Res.	No hyperbaric oxygen therapy
Laubach et al	2018	Hautarzt.	No hyperbaric oxygen therapy
Kanji et al	2017	Mediators Inflamm.	No hyperbaric oxygen therapy
Walter et al	2014	Eur J Clin Mircobiol Infect Dis.	No hyperbaric oxygen therapy
Patel et al	2012	Plast Reconstr Surg.	No hyperbaric oxygen therapy
Zhu et al	2011	Med Hypotheses.	No hyperbaric oxygen therapy
Moen et al	2012	BMC Cancer.	No hyperbaric oxygen therapy
Vescovi P et al	2010	Minerva Stomatol.	No hyperbaric oxygen therapy
Borendal Wodlin et al	2011	BJOG.	No hyperbaric oxygen therapy
Palaniswamy C et al	2011	Clin Cardiol.	No hyperbaric oxygen therapy

Hirn M et al	1989	Ann Chir Gynecol.	No hyperbaric oxygen therapy
Fletcher et al	1972	Am J Roentgenol Radium Ther Nucl Med.	No hyperbaric oxygen therapy
Barber et al	1970	Minn Med.	No hyperbaric oxygen therapy
Krochak et al	1986	Gynecol Oncol.	No hyperbaric oxygen therapy
Puckett et al	2017	Cureus.	No hyperbaric oxygen therapy
Thompson et al	2013	J Neurotrauma.	No hyperbaric oxygen therapy
Oines et al	2014	World J Gastoenterol.	No hyperbaric oxygen therapy
Mitrovic et al	2006	Bosn J Basic Med Sci.	Other comparison
Hu et al	2008	Acta Neurochir Suppl.	Other comparison
Takeyama et al	2007	Knee Surg Sports Traumatol Artrosc.	Other comparison
Van Voorhis et al	2005	Fertil Steril.	Other comparison
Leverment et al	2004	Undersea Hyperb Med.	Other comparison
MacKenzie et al	2003	Transplant Proc.	Other comparison
Dimitrijevich et al	1999	Wound Reair Regen.	Other comparison
Zhou et al	2015	Neurocrit Care.	Other comparison
Assali et al	1968	Circ Res.	Other comparison
Perrin et al	1970	Am J Obstet Gynecol.	Other comparison
Sokolova et al	1980	Akush Ginekol (Mosk).	Other comparison
Choo-Kang et al	1999	J Pediatr Gastoenterol Nutr.	Other comparison
Dische et al	1999	Radiother Oncol.	Other outcome measure
No authors	1978	Lancet.	Other outcome measure
Watson et al	1978	Br J Radiol.	Other outcome measure
Dische et al	1983	Br J Radiol.	Other outcome measure
Ward et al	1979	Clin Radiol.	Other outcome measure
Cade et al	1978	Clin Radiol.	Other outcome measure

Brady et al	1981	Int J Radiat Oncol Biol Phys.	Other outcome measure
Dische et al	1974	Br J Radiol.	Other outcome measure
Ward et al	1974	Br J Radiol.	Other outcome measure
Johnson et al	1974	Am J Roentgenol Radium Ther Nucl Med.	Other outcome measure
Nikitina et al	1978	Vopr Onkol.	Other outcome measure
Smith et al	1970	Br J Radiol.	Other outcome measure
Bewley et al	1970	Br J Radiol.	Other outcome measure
Bates et al	1969	Br J Radiol.	Other outcome measure
Dobrotin et al	1973	Sov Med.	Other outcome measure
Zel’vin et al	1979	Vopr Onkol.	Other outcome measure
Fletcher et al	1977	Cancer.	Other outcome measure
Liu et al	2021	Adv Sci (Weinh).	Other outcome measure
Alagoz et al	1995	Cancer.	Other research question
Hirst et al	1986	Int J Radiat Oncol Biol Phys.	Other research question
Migliorati et al	2005	Cancer.	Other research question
Gaffney et al	2014	Int J Gynecol Cancer.	Other research question
Bennett et al	2012	Cochrane Database Syst Rev.	Other research question
Li et al	2018	ACS Appl Mater Interfaces.	Other research question
Ogawa et al	2013	Int J Clin Oncol.	Other research question
Sirin et al	2011	Int J Med Sci.	Other research question
Wang et al	2011	Spine (Phila Pa 1976).	Other research question
Cummins et al	2010	J Cardiovasc Transl Res.	Other research question
Niu et al	2011	J Orthop Res.	Other research question
Jan et al	2010	Oral Surg Oral Med Oral Pathol Oral Radiol Endod.	Other research question
Moen et al	2009	BMC Cancer.	Other research question

Messier et al	1990	Undersea Biomed Res.	Other research question
Selvendiran et al	2010	Cancer Biol Ther.	Other research question
Friedman et al	2003	Ann Plat Surg.	Other research question
Monies-Chass et al	1975	J Laryngol Otol.	Other research question
Blanchette et al	2014	Hawaii J Med Public Health.	Other research question
Johnson et al	1979	Int J Radiat Oncol Biol Phys.	Other research question
Curet et al	1971	Am J Obstet Gynecol.	Other research question
Dische et al	1980	Br J Radiol.	Other research question
Grant W 3rd et al	1975	Br J Radiol.	Other research question
Brady et al	1976	Cancer.	Other research question
Greenberger et al	2001	Antioxid Redox Signal.	Other research question
Bornstein et al	1964	Medicine (Baltimore).	Other research question
Chiang et al	2016	Int J Med Sci.	Other research question
Daraei et al	2014	Am J Otolaryngol.	Other research question
Pontonnier et al	1970	Bull Fed Soc Gynecol Obstet Lang Fr.	Other research question
Ersan et al	1995	Langenbecks Arch Chir.	Other research question
Atkins et al	1965	Am J Roentgenol Radium Ther Nucl Med.	Other research question
Dar’ialova et al	1979	Akush Ginekol (Mosk).	Other research question
Dische et al	1980	Br J Cancer Suppl.	Other research question
Shuvaeva et al	1986	Sov Med.	Other research question
Porai-Koshits et al	1974	Akush Ginekol (Mosk).	Other research question
Percival et al	1974	Proc R Soc Med.	Other research question
Lu et al	2015	J Oral Maxillofac Surg.	Other research question
Zwaans et al	2018	Int Urol Nephrol.	Other research question
Jia et al	2020	Gynecol Oncol.	Other research question

JÃ°nior et al	2020	Radiat Oncol.	Other research question
Silva et al	2021	Connect Tissue Res.	Other research question
Cooper et al	2021	StatPearls [Internet].	Other research question
Liao et al	2020	Clin Oral Investig.	Other research question
Yuan et al	2020	Int J Mol Sci.	Other research question
Engel et al	2020	Clin Hemorheol Microcirc.	Other research question
Kruize et al	2020	Dermatol Surg.	Other research question
Wang et al	2020	Biomater Sci.	Other research question
Lin et al	2019	Osteoarthritis Cartilage.	Other research question
Reischl et al	2021	Langenbecks Arch Surg.	Other research question
Bennett et al	2005	Cochrane Database Syst Rev.	Other research question
Miles et al	2014	Cochrane Database Rev.	Systematic review
Denton et al	2003	Cochrane Database Syst Rev.	Systematic review
Bennett et al	2018	Cochrane Database Syst Rev.	Systematic review
Allen et al	2012	Support Care Cancer.	Systematic review
Bennett et al	2008	Cancer Treat Rev.	Systematic review
Yuan et al	2020	Front Oncol.	Systematic review
Lopes et al	2018	Undersea Hyperb Med.	Vulvar resection
