# Supplementary material for: Hyperbaric oxygen treatment for late radiation-induced tissue toxicity in treated gynaecological cancer patients: a systematic review
Source: Radiat Oncol. 2022 Oct 6;17:164. doi: 10.1186/s13014-022-02067-6 (PMC9540739; doi:10.1186/s13014-022-02067-6)
Supplement: Supplementary file 5 — Additional file 5. Table 8. Reasons of exclusion from Embase search. [file 13014_2022_2067_MOESM5_ESM.pdf]

Table 8. Reasons of exclusion from Embase search

Author(s)	Year	Journal abbreviation	Reason of exclusion
Maher et al	2007	Clin Oncol.	Article not available
Tewari et al	2002	J Obstet Gynaecol Res.	Article not available
Devita et al	1999	Cancer J Sci Am.	Article not available
No authors	1997	Consultant.	Article not available
Yusa et al	1986	Hiroshima J Anesth.	Article not available
Vigliotti et al	1986	Clin Obstet Gynecol.	Article not available
Rasstrigin et al	1985	Anesteziol Reanimatol.	Article not available
Plenk et al	1981	Int J Radiat Oncol Biol Phys.	Article not available
Kiseleva et al	1981	Med Radiol.	Article not available
Dische et al	1979	Br J Radiol.	Article not available
Ward et al	1978	Br J Radiol.	Article not available
Vagner et al	1977	Vopr Onkol.	Article not available
Arimura et al	1976	Acta Obstet GYnecol Jpn.	Article not available
Bolla et al	1975	Rev Med Alpes Fr.	Article not available
Hamada et al	1971	Gan No Rinsho.	Article not available
Zebro et al	1969	Patol Pol.	Article not available
Masari et al	2019	Int J Gynecol Cancer.	Case report
Qiao et al	2019	Int J Urol.	Case report
Koh et al	2017	BJU Int.	Case report
Fumihiro et al	2017	J Obstet Gynecol Res.	Case report
Costa et al	2015	Int J Gynecol Cancer.	Case report
McDonald et al	2015	Am J Gastroenterol.	Case report
Hayashi et al	2014	J Med Case Rep.	Case report

Geisler et al	2008	J Reprod Med.	Case report
Micha et al	2008	J Reprod Med.	Case report
Huddy et al	2006	Eur J Gastroenterol Hepatol.	Case report
Wang et al	1999	Eur J Obstet Gynecol Reprod Biol.	Case report
Ishibashi et al	1996	Jpn J Urol.	Case report
Hamour et al	1996	Lancet.	Case report
Schoenrock et al	1986	Urology.	Case report
Kaercher et al	1975	Wien Klin Wochenschr.	Case report
Yoshimizu et al	2017	Dig Endosc.	Case series
Terada et al	2003	Nishinihon J Urol.	Case series
Lee et al	1994	Undersea Hyperb Med.	Case series
Weiss et al	1989	J Urol.	Case series
Miyazato et al	1998	Jpn J Urol.	Language
Bates et al	1974	Br J Radiol.	Letter
Yip et al	2013	Ther Adv Urol.	No gynaecological malignancies
Adachi et al	2013	Mol Clin Oncol.	No gynaecological malignancies
Oronsky et al	2011	Transl Oncol.	No gynaecological malignancies
Degener et al	2015	BMC Urol.	No gynaecological malignancies
Neheman et al	2005	BJU Int.	No gynaecological malignancies
Cagetti et al	2021	Gynecol Oncol.	No hyperbaric oxygen therapy
Ortiz et al	2021	Curr Neuropharmacol.	No hyperbaric oxygen therapy
Chapman et al	2020	Gynecol Oncol.	No hyperbaric oxygen therapy
Hernandez et al	2020	BMJ Case Rep.	No hyperbaric oxygen therapy
Männle et al	2020	Onkologe.	No hyperbaric oxygen therapy
Tanaka et al	2019	JA Clin Rep.	No hyperbaric oxygen therapy

Goucher et al	2019	Can Urol Assoc J.	No hyperbaric oxygen therapy
Wang et al	2019	Cancers.	No hyperbaric oxygen therapy
Lobo et al	2018	Urology.	No hyperbaric oxygen therapy
Ohno et al	2018	Cancers.	No hyperbaric oxygen therapy
Wang et al	2018	Trends Pharmacol Sci.	No hyperbaric oxygen therapy
Natesan et al	2016	Int J Gynecol Cancer.	No hyperbaric oxygen therapy
Curtis et al	2016	Crit Rev Oncol Hematol.	No hyperbaric oxygen therapy
Sanders et al	2016	Am J Otolaryngol.	No hyperbaric oxygen therapy
Murakami et al	2016	Anticancer Res.	No hyperbaric oxygen therapy
Lewis et al	2016	Brachytherapy.	No hyperbaric oxygen therapy
Hill et al	2015	Semin Radiat Oncol.	No hyperbaric oxygen therapy
Gupta et al	2015	Am J Case Rep.	No hyperbaric oxygen therapy
Murakami et al	2014	Radiat Oncol.	No hyperbaric oxygen therapy
D'Souza et al	2014	Brachytherapy.	No hyperbaric oxygen therapy
Vargo et al	2014	Radiother Oncol.	No hyperbaric oxygen therapy
Busk et al	2013	Q J Nucl Med Mol Imaging.	No hyperbaric oxygen therapy
Jensen et al	2013	Int J Gynecol Cancer.	No hyperbaric oxygen therapy
Sahakitrungruang et al	2012	Dis Colon Rectum.	No hyperbaric oxygen therapy
Maccio et al	2012	Cytokine.	No hyperbaric oxygen therapy
Ho et al	2009	Malays J Med Sci.	No hyperbaric oxygen therapy
Gerber et al	2008	Am Fam Physician.	No hyperbaric oxygen therapy
Adamski et al	2008	Cancer Treat Rev.	No hyperbaric oxygen therapy
Groenman et al	2008	J Minim Invasive Gynecol.	No hyperbaric oxygen therapy
Robinson et al	2008	Medicine.	No hyperbaric oxygen therapy
Van den Wyngaert et al	2006	Ann Oncol.	No hyperbaric oxygen therapy

Sharma et al	2005	J Indian Acad Clin Med.	No hyperbaric oxygen therapy
Powell et al	2003	J Pelvic Med Surg.	No hyperbaric oxygen therapy
Martin-Loeches et al	2003	Arch Gynecol Obstet.	No hyperbaric oxygen therapy
Levenback et al	1995	Clin Cons Obstet Gynecol.	No hyperbaric oxygen therapy
Alvaro-Villegas et al	2011	Rev Esp Enferm Dig.	Other comparison
Alvaro-Villegas et al	2010	Gastrointest Endosc.	Other comparison
Ward et al	1979	Clin Radiol.	Other outcome measure
Zelvin et al	1979	Vopr Onkol.	Other outcome measure
Watson et al	1978	Br J Radiol.	Other outcome measure
Dische et al	1978	Br J Radiol.	Other outcome measure
Bush et al	1978	Lancet.	Other outcome measure
Cade et al	1978	Clin Radiol.	Other outcome measure
Fletcher et al	1977	Cancer.	Other outcome measure
Ward et al	1974	Br J Radiol.	Other outcome measure
Dische et al	1974	Br J Radiol.	Other outcome measure
Johnson et al	1974	Am J Roentgenol Radium Ther Nucl Med.	Other outcome measure
Dische et al	1983	Br J Radiol.	Other outcome measure
Nikitina et al	1978	Vopr Onkol.	Other outcome measure
Chapman et al	2021	Brachytherapy.	Other research question
Jethwa et al	2020	J Radiat Oncol.	Other research question
Shejul et al	2020	Radiother Oncol.	Other research question
Mulvihill et al	2020	Birth Defects Res.	Other research question
Song et al	2020	J Cancer Res Clin Oncol.	Other research question
Bosch et al	2020	Neurourol Urodyn.	Other research question
Klement et al	2020	Expert Rev Anticancer Ther.	Other research question

Kim et al	2020	J Gynecol Oncol.	Other research question
Murofushi et al	2020	Int J Gynecol Cancer.	Other research question
Skwarski et al	2020	Cancer Drug Discov Dev.	Other research question
Forster et al	2019	Phys Med.	Other research question
Waisaing et al	2019	Int J Urol.	Other research question
Zulkifle et al	2019	BMJ Case Rep.	Other research question
Zwaans et al	2018	Int Urol Nephrol.	Other research question
Bennett et al	2018	Diving Hyperb Med.	Other research question
Fernandes et al	2015	Curr Opin Oncol.	Other research question
Liang et al	2015	Mol Med Rep.	Other research question
Heyboer et al	2014	Stem Cell Res.	Other research question
Gaffney et al	2014	Int J Gynecol Cancer.	Other research question
Marson et al	2014	Current Bladder Dysfunct Rep.	Other research question
Vargo et al	2014	Brachytherapy.	Other research question
Vargo et al	2013	Brachytherapy.	Other research question
Broderick et al	2012	BJOG: Int J Obstet Gynecol.	Other research question
Bennett et al	2012	Cochrane Database Syst Rev.	Other research question
Azeem et al	2012	Am J Clin Oncol: Cancer Clin Trials.	Other research question
Chino et al	2010	Dig Endosc.	Other research question
Jensen et al	2010	Brachytherapy.	Other research question
Al-Waili et al	2005	Med Sci Monit.	Other research question
Varlotto et al	2005	Int J Radiat Oncol Biol Phys.	Other research question
Migliorati et al	2005	Cancer.	Other research question
Harrison et al	2002	Oncologist.	Other research question
Coleman et al	2002	J Clin Oncol.	Other research question

Grigsby et al	1999	Cancer Control.	Other research question
Pomeroy et al	1998	J Urol.	Other research question
Miura et al	1996	Int Urol Nephrol.	Other research question
Alagoz et al	1995	Cancer.	Other research question
Teicher et al	1995	Hematol Oncol Clin North Am.	Other research question
Thomas et al	1994	Semin Radiat Oncol.	Other research question
Eifel et al	1993	Gynecol Oncol.	Other research question
Searly et al	1989	Cancer.	Other research question
Hirst et al	1986	Int J Radiat Oncol Biol Phys.	Other research question
Dische et al	1980	Br J Radiol.	Other research question
Johnson et al	1979	Int J Radiat Oncol Biol Phys.	Other research question
Fryer et al	1979	Br J Radiol.	Other research question
Glassburn et al	1977	Cancer.	Other research question
Brady et al	1976	Cancer.	Other research question
Volkova et al	1976	Med Radiol.	Other research question
Wiernik et al	1975	Br J Radiol.	Other research question
Grant III et al	1975	Br J Radiol.	Other research question
Rounthwaite et al	1968	Laryngoscope.	Other research question
Ogawa et al	2013	Int J Clin Oncol.	Other research question
McLaughlin et al	2000	Cancer Res Ther Control.	Other research question
Dische et al	1999	Radiother Oncol.	Other research question
Noordzij et al	1993	Int Urogynecol J.	Other research question
Fernández et al	2021	Clin Transl Oncol.	Review article
Creutzberg et al	2016	Lancet Oncol.	Review article
Feldmeier et al	2011	Curr Oncol.	Review article

Datta et al	2018	Strahlenther Onkol.	Systematic review
Moen et al	2012	Target Oncol.	Systematic review
Allen et al	2012	Support Care Cancer.	Systematic review
Bennett et al	2008	Cancer Treat Rev.	Systematic review
Daruwalla et al	2006	World J Surg.	Systematic review
Lopes et al	2016	Int J Gynecol Cancer.	Vulvar resection
