# Supplementary material for: Hyperbaric oxygen treatment for late radiation-induced tissue toxicity in treated gynaecological cancer patients: a systematic review
Source: Radiat Oncol. 2022 Oct 6;17:164. doi: 10.1186/s13014-022-02067-6 (PMC9540739; doi:10.1186/s13014-022-02067-6)
Supplement: Supplementary file 6 — Additional file 6. Table 9. Reasons of exclusion from the Cochrane library search. [file 13014_2022_2067_MOESM6_ESM.pdf]

Table 9. Reasons of exclusion from the Cochrane library search

Author(s)	Year	Journal abbreviation	Reason of exclusion
Plenk et al	1981	Int J Radiat Oncol Biol Phys.	Article not available
Plenk et al	1972	Am J Roentgenol Radium Ther Nucl Med.	Article not available
Tobin et al	1971	Am J Roentgenol Radium Ther Nucl Med.	Article not available
No authors	Not published yet	Not published yet	Not published yet
Ward et al	1979	Clin Radiol.	Other outcome measure
Brady et al	1981	Int J Radiat Oncol Biol Phys.	Other outcome measure
Ward et al	1974	Br J Radiol.	Other outcome measure
Dische et al	1974	Br J Radiol.	Other outcome measure
Cade et al	1978	Clin Radiol.	Other outcome measure
Fletcher et al	1977	Cancer.	Other outcome measure
Dische et al	1999	Radiother Oncol.	Other outcome measure
Watson et al	1978	Br J Radiol.	Other outcome measure
Dische et al	1980	Br J Radiol.	Other research question
Shao et al	2012	BJU Int.	Other research question
Van de Wetering et al	2016	Cochrane Database Syst Rev.	Systematic review
Bennett et al	2018	Cochrane Database Syst Rev.	Systematic review
Miles et al	2014	Cochrane Database Syst Rev.	Systematic review
Denton et al	2003	Cochrane Database Syst Rev.	Systematic review
