# Supplementary material for: Hyperbaric oxygen treatment for late radiation-induced tissue toxicity in treated gynaecological cancer patients: a systematic review
Source: Radiat Oncol. 2022 Oct 6;17:164. doi: 10.1186/s13014-022-02067-6 (PMC9540739; doi:10.1186/s13014-022-02067-6)
Supplement: Supplementary file 7 — Additional file 7. Table 10. Study design of the included studies. [file 13014_2022_2067_MOESM7_ESM.pdf]

Table 10. Study design of the included studies

Author, year	Title	Journal abbreviation	Study design
Oscarsson et al, 2013 [14]	Hyperbaric oxygen treatment in radiation-induced cystitis and proctitis: a prospective cohort study on patient-perceived quality of recovery.	Int J Radiat Oncol Biol Phys.	Prospective cohort study
Glover et al, 2016 [15]	Hyperbaric oxygen for patients with chronic bowel dysfunction after pelvic radiotherapy (HOT2): a randomised, double-blind, sham-controlled phase 3 trial.	Lancet Oncol.	Randomised controlled trial
Oscarsson et al, 2019 [16]	Radiation-induced cystitis treated with hyperbaric oxygen therapy (RICH-ART): a randomised, controlled, phase 2-3 trial.	Lancet Oncol.	Randomised controlled trial
Oliai et al, 2012 [17]	Hyperbaric oxygen therapy for radiation-induced cystitis and proctitis.	Int J Radiat Oncol Biol Phys.	Retrospective study
Sidik et al, 2007 [18]	Does hyperbaric oxygen administration decrease side effect and improve quality of life after pelvic radiation?	Acta Med Indones.	Open randomized controlled trial
Clarke et al, 2008 [19]	Hyperbaric oxygen treatment of chronic refractory radiation proctitis: a randomized and controlled double-blind crossover trial with long-term follow-up.	Int J Radiat Oncol Biol Phys.	Randomised controlled double-blind crossover trial
Parra et al, 2011 [20]	[Management of hemorrhagic radiation cystitis with hyperbaric oxygen therapy].	Actas Urol Esp.	Retrospective study

Rud et al, 2009 [21]	Hyperbaric oxygen therapy for late radiation tissue injury in gynaecological patients.	Support Care Cancer.	Prospective study
Safra et al, 2008 [22]	Improved quality of life with hyperbaric oxygen therapy in patients with persistent pelvic radiation-induced toxicity.	Clin Oncol (R Coll Radiol).	Retrospective study
Jones et al, 2006 [23]	Treatment of radiation proctitis with hyperbaric oxygen.	Radiother Oncol.	Retrospective study
Williams et al, 1992 [24]	The treatment of pelvic soft tissue radiation necrosis with hyperbaric oxygen.	Am J Obstet Gynecol.	Prospective observational study
Feldmeier et al, 1996 [25]	Hyperbaric oxygen an adjunctive treatment for delayed radiation injuries of the abdomen and pelvis.	Undersea Hyperb Med.	Retrospective analysis
Al-Ali et al, 2010 [26]	Is treatment of hemorrhagic radiation cystitis with hyperbaric oxygen effective?	Urol Int.	Retrospective study
Bui et al, 2004 [27]	The efficacy of hyperbaric oxygen therapy in the treatment of radiation-induced late side effects.	Int J Radiat Oncol Biol Phys.	Retrospective study
Andren et al, 2020 [28]	An observational trial to establish the effect of hyperbaric oxygen treatment on pelvic late radiation injury due to radiotherapy	Diving Hyperb Med.	Combined retrospective and prospective observational study

Ngoo et al, 2018 [29]	The utility of hyperbaric oxygen therapy in managing haemorrhagic radiation-induced cystitis	Int J Urol.	Retrospective study
Lin et al, 2017 [30]	Hyperbaric oxygen therapy for hemorrhagic radiation cystitis	Formos J Surg.	NR
Ribeiro de Oliveira et al, 2015 [31]	Hyperbaric oxygen therapy for refractory radiation-induced hemorrhagic cystitis	Int J Urol.	Retrospective study
Mougin et al, 2016 [32]	Evaluation of hyperbaric oxygen therapy in the treatment of radiation-induced hemorrhagic cystitis	Urology.	Retrospective study
Ferreira et al, 2014 [33]	Hyperbaric oxygen for long-term complications of radiation cystitis	J Radiother Pract.	Retrospective study
Fink et al, 2006 [34]	Hyperbaric oxygen therapy for delayed radiation injuries in gynecological cancers	Int J Gynecol Cancer.	Retrospective study
