# Supplementary material for: Hyperbaric oxygen treatment for late radiation-induced tissue toxicity in treated gynaecological cancer patients: a systematic review
Source: Radiat Oncol. 2022 Oct 6;17:164. doi: 10.1186/s13014-022-02067-6 (PMC9540739; doi:10.1186/s13014-022-02067-6)
Supplement: Supplementary file 8 — Additional file 8. Table 11. Time. [file 13014_2022_2067_MOESM8_ESM.pdf]

Table 11. Time

Study, year	Time radiation to injury (years)	Time injury to therapy (years)	Follow-up (months)
Oscarsson et al, 2013 [14]	Urinary symptoms 1.5 (mean)<br>Bowel symptoms mean 0.5 (mean)	NR	6-12
Glover et al, 2016 [15]	Treatment 3.5 (IQR 2.3–9.7)<br>Control 3.9 (IQR 2.5-5.7)	NR	13.2 (median)
Oscarsson et al, 2019 [16]	Treatment 4.4^{a} (SD 5.1)<br>Control 4.1^{a} (SD 3.4)	Treatment 3.1 (SD 4.8)<br>Control 2.8 (SD 2.8)	6-8
Oliai et al, 2012 [17]	Cystitis 2.5 (median)<br>Proctitis 0.9 (median)	NR	39 (median)
Sidik et al, 2007 [18]	NR	NR	6
Clarke et al, 2008 [19]	1.5 * (mean)	NR	12-60
Parra et al, 2011 [20]	2.6 (mean)	0.4 (mean)	21.2 (mean)
Rud et al, 2009 [21]	3.5^{a} (median)	3.5^{a} (median)	6
Safra et al, 2008 [22]	2.7^{a} (mean)	2.7^{a} (mean)	NR
Jones et al, 2006 [23]	NR	1.7 (mean)	25 (median)
Williams et al, 1992 [24]	NR	NR	NR

Feldmeier et al, 1996 [25]	8.3 * (mean)	2.8 * (mean)	NR
Al-Ali et al, 2010 [26]	NR	0.7 (mean)	18 (mean)
Bui et al, 2004 [27]	NR	NR	NR
Andren et al, 2020 [28]	NR	NR	NR
Ngoo et al, 2018 [29]	1.4	0.4 (median)	5.1 (median)
Lin et al, 2017 [30]	9.9 (mean)	NR	20.7 (mean)
Ribeiro de Oliveira et al, 2015 [31]	4.6 (mean)	1.1 (mean)	12 (mean)
Mougin et al, 2016 [32]	3.2 (median)	0.7 (mean)	15 (median)
Ferreira et al, 2014 [33]	2.3 (median)	0.7 (median)	55.5 (median)
Fink et al, 2006 [34]	1.7 (mean)	NR	32.5 (mean)

a: time from radiation to HBOT in years

NR = not reported

SD = standard deviation

IQR = interquartile range

* = calculated time of only the gynaecological malignancies
