# Supplementary material for: Hyperbaric oxygen treatment for late radiation-induced tissue toxicity in treated gynaecological cancer patients: a systematic review
Source: Radiat Oncol. 2022 Oct 6;17:164. doi: 10.1186/s13014-022-02067-6 (PMC9540739; doi:10.1186/s13014-022-02067-6)
Supplement: Supplementary file 9 — Additional file 9. Table 12. Reported side effects. [file 13014_2022_2067_MOESM9_ESM.pdf]

Table 12. Reported side effects

Study, year	Reported side effects (number of patients)
Oscarsson et al, 2013 [14]	No side effects
Glover et al, 2016 [15]	Eye refractive change including myopia (n=19)<br>Increased fatigue or tiredness (n=5)<br>Ear pain or barotrauma (n=21)<br>Tonsillitis grade 3 (n=1)<br>Recurrent cancer of the vulva grade 4 (n=1)<br>Malignant spinal cord compression grade 3 (n=1)<br>Malignant paraortic lymph node involvement grade 3 (n=1)<br>Recurrence of vomiting and dehydration grade 3 (n=1)<br>Diarrhoea and fever associated with campylobacter infection grade 3 (n=1)<br>Recurrence of abdominal pain, bloating, diarrhea, urinary tract infection grade 3 (n=1)<br>Aneurysma grade 4 (n=1)
Oscarsson et al, 2019 [16]	Temporary pain in the ear (n=6)<br>Barotrauma (n=4)<br>Paracentesis of the tympanic membrane (n=1)<br>Hyperoxia-induced transient myopia with changes in vision (n=5)<br>Induced transient myopia (n=1)<br>Panic attack related to pre-existing claustrophobia (n=1)<br>Fatigue grade 1 (n=1)<br>Infections grade 1 and grade 3 (n=7)<br>Transient muscle cramps or pain grade 1 (n=5)<br>Haematuria grade 1 and grade 3 (n=3)<br>Diarrhoea grade 1 (n=2)<br>Nausea grade 1 (n=1)<br>Headache grade 1 (n=1)<br>Death due to sepsis and cardiac failure unrelated to the study (n=1)
Oliai et al, 2012 [17]	Middle ear equilization changes manifesting as otalgia (n= 5)

Sidik et al, 2007 [18]	NR
Clarke et al, 2008 [19]	Ear pain or discomfort (n=11)<br>Tympanic membrane changes consistent with barotrauma (n=7)<br>Tympanic membrane injury and middle ear effusion (n=1)<br>Sinus barotrauma (n=1)<br>Transient myopia (n=4)<br>Confinement anxiety (n=2)
Parra et al, 2011 [20]	Barotraumatic otitis (n=2)
Rud et al, 2009 [21]	NR
Safra et al, 2008 [22]	No side effects
Jones et al, 2006 [23]	Minor otic barotrauma (n=4)<br>Respiratory compromise (n=1)
Williams et al, 1992 [24]	Ruptured tympanum (n=1)
Feldmeier et al, 1996 [25]	Persistent tumor and subsequently death (n=1)<br>Gastrointestinal bleed and cardiac deterioration (n=1)<br>Progressive necrosis of many pelvic viscera and died of complications secondary to radiation injury (n=1)
Al-ali et al, 2010 [26]	No side effects
Bui et al, 2004 [27]	Hearing (n=10): hearing difficulties to ear pain and one had serious effusion in one ear<br>Vision (n=3): one short-lived lens swelling and two accelerated cataract formation<br>Epistaxis (n=1)
Andren et al, 2020 [28]	Ear pain (n=11)<br>Barotrauma (n=2)<br>Myopia (n=4)
Ngoo et al, 2018 [29]	Barotrauma (n=2)
Lin et al, 2017 [30]	No side effects
Ribeiro de Oliveira et al, 2015 [31]	Mild middle ear barotrauma (n=3)
Mougin et al, 2016 [32]	Barotitis media (n=9)<br>Vision disorder (n=5)<br>Finger paresthesia (n=1)

	Pain during mobilization (n=1)<br>Discontinuation for adverse events (n=2)
Ferreira et al, 2014 [33]	Barotraumatic otitis (n=3)
Fink et al, 2006 [34]	Oxygen-toxic seizure (n=1)<br>Temporarily ear equilization problems or mild barotrauma (n=2)

NR= not reported
